# Recent progress of exciton transport in two-dimensional semiconductors

**DOI:** 10.1186/s40580-023-00404-3

**Published:** 2023-12-15

**Authors:** Hyeongwoo Lee, Yong Bin Kim, Jae Won Ryu, Sujeong Kim, Jinhyuk Bae, Yeonjeong Koo, Donghoon Jang, Kyoung-Duck Park

**Affiliations:** Department of Physics, Pohang University of Science and Technology (POSTECH), Pohang, 37673 Republic of Korea

**Keywords:** Two-dimensional semiconductors, Electrical control, Strain gradient, Surface plasmon polaritons, Photonic cavity, Exciton transport

## Abstract

Spatial manipulation of excitonic quasiparticles, such as neutral excitons, charged excitons, and interlayer excitons, in two-dimensional semiconductors offers unique capabilities for a broad range of optoelectronic applications, encompassing photovoltaics, exciton-integrated circuits, and quantum light-emitting systems. Nonetheless, their practical implementation is significantly restricted by the absence of electrical controllability for neutral excitons, short lifetime of charged excitons, and low exciton funneling efficiency at room temperature, which remain a challenge in exciton transport. In this comprehensive review, we present the latest advancements in controlling exciton currents by harnessing the advanced techniques and the unique properties of various excitonic quasiparticles. We primarily focus on four distinct control parameters inducing the exciton current: electric fields, strain gradients, surface plasmon polaritons, and photonic cavities. For each approach, the underlying principles are introduced in conjunction with its progression through recent studies, gradually expanding their accessibility, efficiency, and functionality. Finally, we outline the prevailing challenges to fully harness the potential of excitonic quasiparticles and implement practical exciton-based optoelectronic devices.

## Introduction

Excitonic quasiparticles play a dominant role in the distinctive optical characteristics of two-dimensional (2D) semiconductors [1–9]. These characteristics arise from their direct bandgap, quantum confinement effect, strong light-matter interaction, and valley-specific dynamics [10–12]. The ability to spatially control excitonic quasiparticles offers exciting prospects for developing quantum information devices and exciton-integrated circuits [13, 14]. Excitons can substitute the conventional electron-based carriers with an advantage of much less thermal energy loss. Furthermore, enhanced controllability of excitonic quasiparticles holds promise for achieving high-efficiency energy harvesting in excitonic photovoltaics [15] and quantum light-emitting systems [16, 17]. However, the distribution of excitonic quasiparticles in 2D area has posed challenges due to the lack of electrical controllability for neutral excitons, the short lifetime of charged excitons [18], and the low exciton funneling efficiency at room temperature [15, 19]. Although various approaches have been explored to modify the control methods and alter the species of excitonic quasiparticles, complete spatial control over long-range exciton transport remains elusive.

**Fig. 1:**
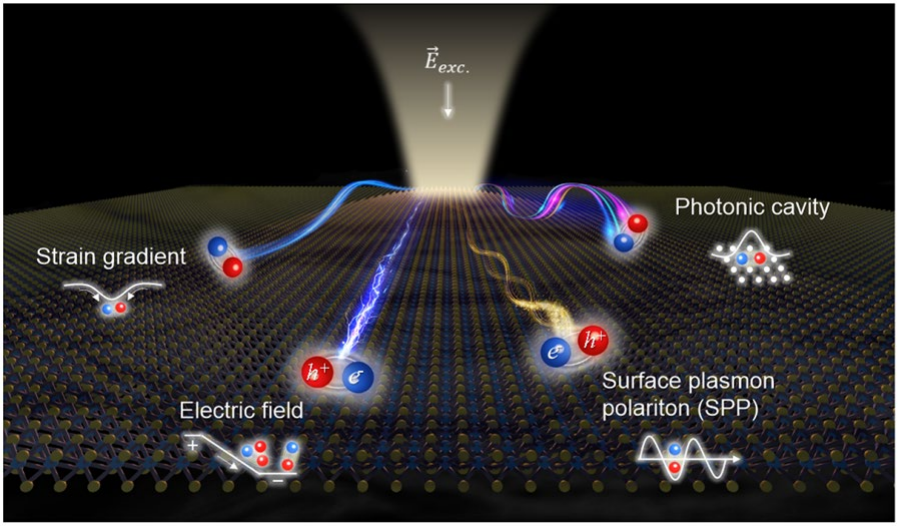
Illustration of four different control methods of exciton transport in two-dimensional semiconductors

In this comprehensive review, we present recent advancements for the control of excitonic quasiparticles in 2D semiconductors, as illustrated in Fig. 1. We focus primarily on the progress made in controlling exciton currents using various control parameters, namely electric fields, strain gradients, surface plasmon polaritons (SPPs), and photonic cavities. For each control parameter, we discuss how the unique properties of excitonic quasiparticles, structural advancements, and various control methods have contributed to their technological progress. In part 1, we investigate the electrical control of excitonic quasiparticles, specifically charged excitons and interlayer excitons (IX). The behavior of charged excitons is governed by the electrostatic potential, while IX are influenced by the quantum confined Stark effect. Part 2 highlights the strain control of neutral excitons facilitated by strain gradients in transition metal dichalcogenide (TMD) monolayers transferred on the artificial structures. We explore how the spatial extent of strain gradient plays a crucial role in determining the diffusion and drift characteristics of neutral excitons. Moving on to part 3, the exciton transport as a coupling form with the SPP, exploiting the unique characteristics of plasmons, such as subwavelength local confinement and long propagation length. We delve into the introduction of roughness-induced SPP that enables directionless propagation, preceded by the advancements of optimized plasmonic structures that facilitate directional transport of excitonic emission. Lastly, part 4 focuses on the developments of the exciton transport coupling with specific photonic modes within photonic cavity structures. We examine the aspects of directionality, valley preservation, long-range transport, and deterministic routing.

We envision that this review provides a comprehensive understanding of exciton transport in two-dimensional semiconductors. In addition, by presenting various control modalities along with their specific progressions, we hope to assist readers to predict future directions on exciton transport study.

## Electrical control for exciton transport

**Fig. 2:**
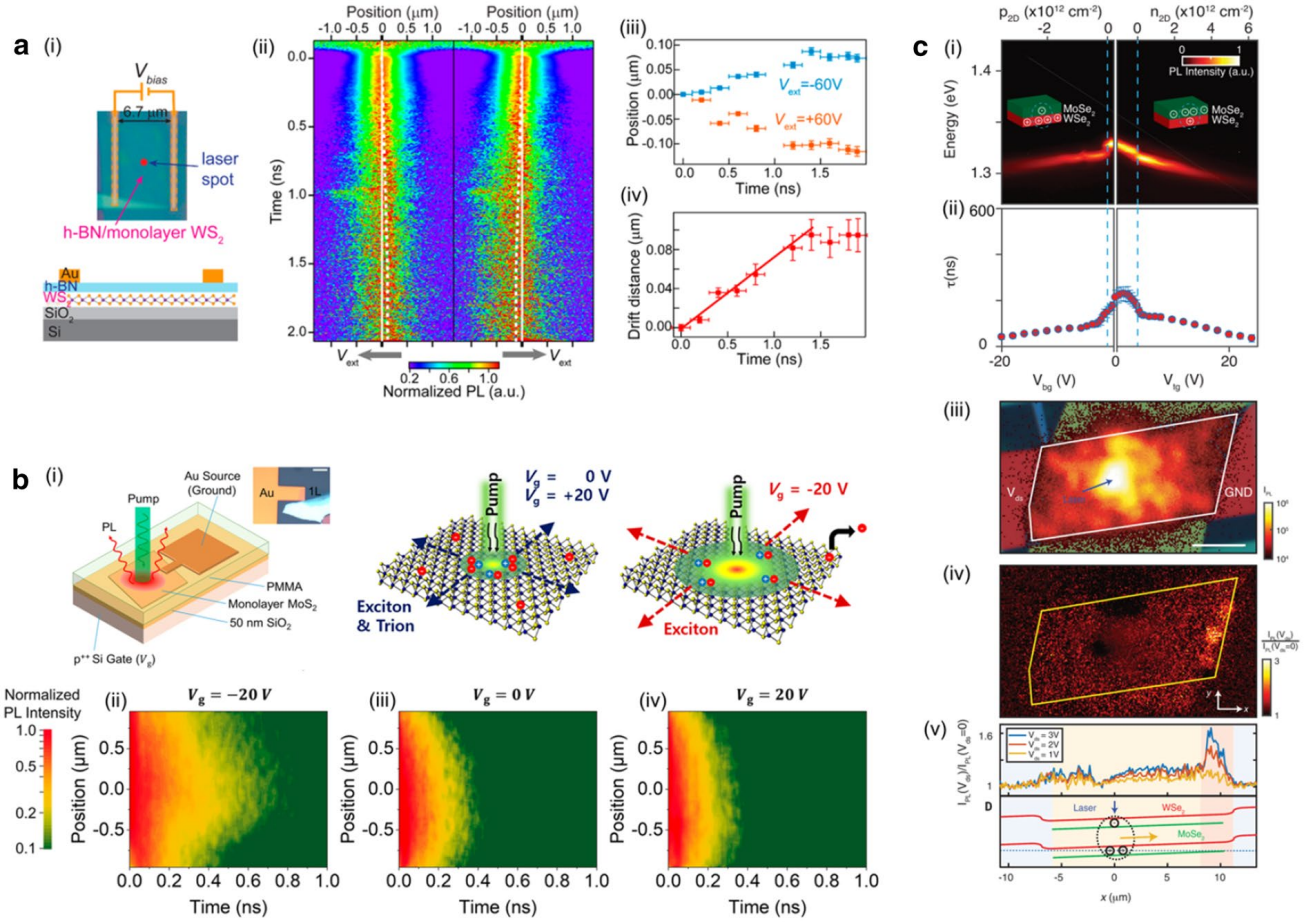
**a** (i) Schematic cross-section view of the h-BN/WS$_2$/SiO$_2$ sample. (ii) Normalized PL images showing the emission of charged excitons under applied bias voltages *V*$_{\text {ext}}$ $=$ +60 V and *V*$_{\text {ext}}$ $=$ −60 V, respectively. (iii) Average shift of the center position of the spatial profiles of the PL emission as a function of decay time at different applied bias voltages. (iv) Drift distance of charged excitons as a function of decay time. **b** (i) Schematic diagram illustrating the generation and behavior of photogenerated neutral and charged excitons in MoS$_2$, depending on gate voltage (*V*$_\text {g}$). Normalized PL images depicting diffusion patterns of neutral and charged excitons at *V*$_\text {g}$ $=$ −20 (ii), 0 (iii), and 20 V (iv), respectively, under a pump fluence of 75 $\mu$J cm$^{-2}$. **c** (i) Single-gate dependence analysis revealing the formation of charged interlayer excitons with varying carrier density. (ii) Decay times for charged interlayer excitons as a function of single gate bias voltage. (iii) Optical image showcasing the device with false-colored top gates covering the overlaid WSe$_2$ and MoSe$_2$ heterostructure. (iv) Spatial dependence of PL intensity normalized according to *I*$_{\text {PL}}$(*V*$_{\text {ds}}$)/*I*$_{\text {PL}}$(*V*$_{\text {ds}}$ $=$ 0) for *V*$_{\text {ds}}$ $=$ −3 V. (v) Normalized *I*$_{\text {PL}}$ averaged over the y axis versus the heterostructure channel length *x* for different *V*$_{\text {ds}}$. **a** Reproduced with permission [20]. Copyright [2021] Americal Chemical Society. **b** Reproduced with permission [18]. Copyright [2020] Americal Chemical Society. **c** Reproduced with permission [21]. Copyright [2019] AAAS

Understanding and manipulating exciton transport in TMD monolayers can lead to the progress of the next generation electronic and optoelectronic device [17, 22–26]. However, the electrical control of the neutral excitons is significantly limited due to their charge neutral property. In contrast to the neutral excitons, the transport of charged excitons, which is a Fermi polaron formed by Coulomb attraction between the exciton and surrounding electrons can be driven by an in-plane electric field [27, 28]. Therefore, charged exciton-based optoelectronic devices are expected with the operation principles analogous to that of the conventional electronic devices.

Recently, according to Cheng et al., the diffusion and the drift of negative charged excitons in monolayer WSe$_2$ was investigated with two Au electrodes with hexagonal boron nitride (h-BN) spacer, as shown in Fig. 2a-(i) [20]. By obtaining the spatially- and temporally-resolved PL response, the spatial profile of charged excitons shifted opposite to the direction of the in-plane electric field, as shown in Fig. 2a-(ii). Figure 2a-(iii) clearly showed the electrically modified distribution of charged excitons under applied bias *V*$_{\text {ext}}$ $=$ 60 V and −60 V. The saturated propagation of charged excitons was observed after $\sim$1.4 ns in both directions of in-plane electric field, corresponds to the transport distance of $\sim$0.1 $\mu$m. This was attributed to the Coulomb attraction between charged excitons and the localized donors, which remained after charged excitons propagate along the opposite direction of the external field. The propagation distance of charged excitons can be controlled by manipulating bias voltage. The propagation distance increased linearly as a function of bias voltage, as shown in Fig. 2a-(iv). These findings clarify the fundamental principles in electrically driven charged exciton transport and paves the way to realize controllable photonic circuit. However, short transport distance of charged excitons in TMD monolayers gives limitation in field of optoelectronic devices.

For the development of charged exciton-based devices, inducing high density of charged excitons is a crucial prerequisite. The study by Uddin et al. presented the generation of charged excitons by adjusting gate voltage on the MoS$_2$ monolayer, as illustrated in Fig. 2b-(i) [18]. At the gate voltage of −20 V, the excess electrons in MoS$_2$ monolayer were moved out to the grounded Au electrode, leading all photo-generated carriers to create neutral excitons. By contrast, at the gate voltage of 20 V, almost all photo-generated carriers turned into negatively charged excitons due to the large concentration of excess electrons. Figure 2b-(ii-iv) showed the spatially- and temporally-resolved distribution of photo-generated excitons with different gate voltages. Charged excitons exhibited shorter diffusion length than neutral excitons, attributed to the difference in their lifetime as charged excitons had shorter lifetime ($\sim$50 ps) compare to neutral excitons ($\sim$10 ns) [29]. In the case of a MoS$_2$ monolayer, they observed that the nonradiative lifetime of trions was significantly shorter than their radiative lifetime by almost three orders of magnitude. This finding strongly suggested that the primary pathway for trion recombination in this system was dominated by nonradiative processes. The observed disparity could potentially arose from either defect-induced decay or an Auger-like mechanism, wherein an electron served as the third particle necessary for momentum conservation. Therefore, while charged excitons facilitates the electrical controllability, their shorter lifetime significantly restricted the transport. Lifetime of excitons can be increased by constructing TMD heterostructures, where spatially separated electrons and holes form IX across the quantum wells [30–36].

Whereas intralayer charged excitons in TMD monolayers exhibit short lifetimes ($\sim$50 ps), the IX within TMD heterostructures manifest significantly extended lifetimes ($\sim$100 ns) [21]. IXs in TMD heterostructures are spatially indirect and momentum-indirect due to the lattice mismatch. Consequently, the phonon-assisted nature of the emission process further reduces the exciton recombination rate, yielding the longer lifetime [37, 38]. Such an extended lifetime can be exploited to obtain the diffusion length of IX over a scale of micrometers, even at room temperature. In the recent study performed by Jauregui et al., the electrostatic condition in TMD heterostructures was well modified by electrostatically doping the one of TMD layers, as shown in Fig. 2c-(i) [21]. The reduced PL energy clearly demonstrated the generation of charged IX [39]. While the lifetime of charged interlayer exciton was $\sim$100 ns near the band edge, it further decreased with increase the charge doping, due to additional decay channel by scattering with free carriers, as shown in Fig. 2c-(ii). Figure 2c-(iii) showed the spatial distribution of photo-generated charged IX with the excitation at the center of the sample, diffusing across the entire sample. To electrically control propagation of charged IX, multiple electrical contacts are attached away from the heterostructure edge. Figure 2c-(iv) showed the change in spatial distribution of charged IX with the existence of the external electric field. When the external electric field was applied across the sample, the charged IX moved opposite to the electric field, as shown in Fig. 2c-(v). Charged IX provide long-range propagation with electrical controllability, clearly resolving the current limitations of neutral intralayer excitons.

**Fig. 3:**
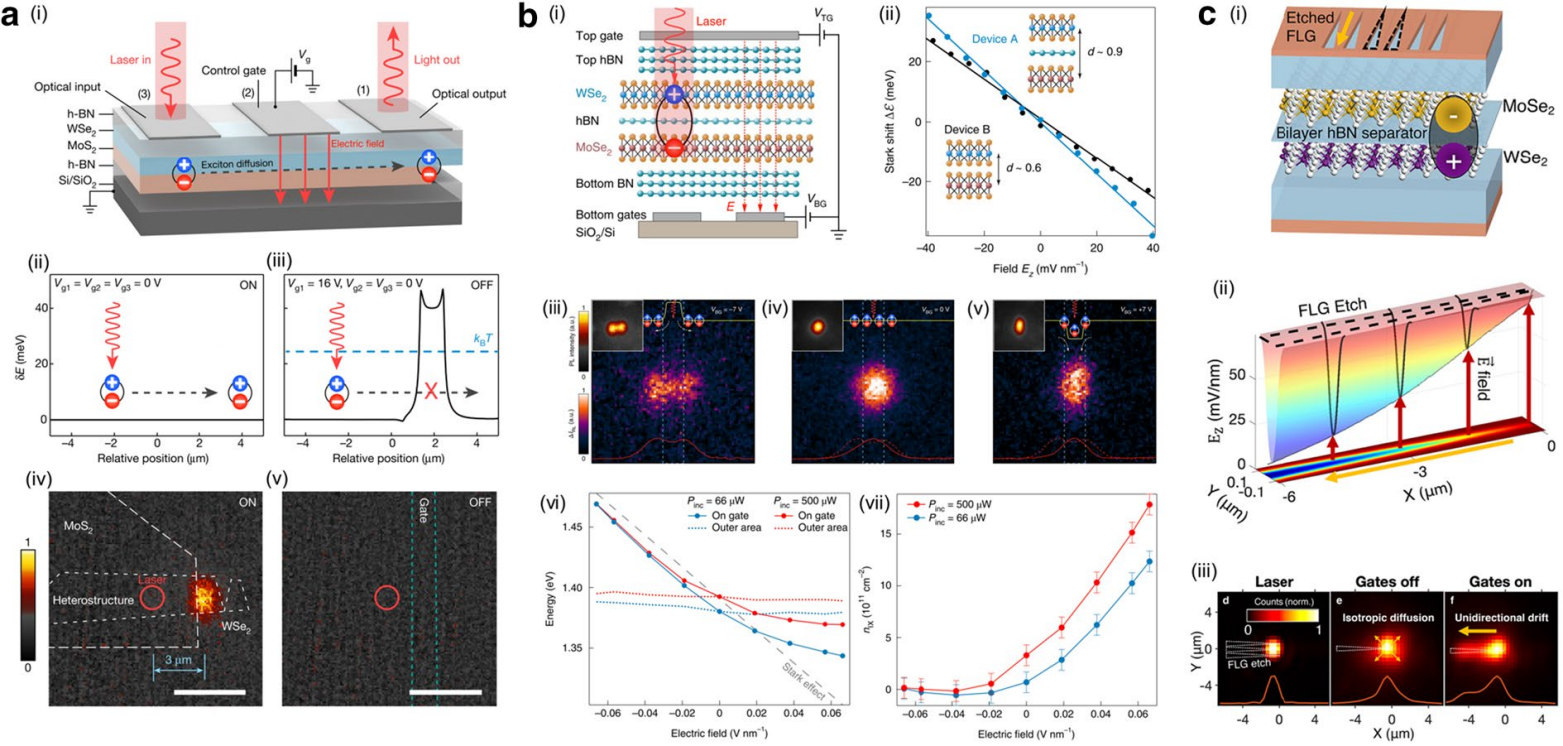
**a** (i) Excitonic transistor designed for engineering the electrostatic potential to control exciton diffusion. Calculated energy variation for the excitons in ON state (free diffusion, ii) and OFF state (potential barrier, iii). Emission images corresponding ON state (free diffusion, iv) and OFF state (potential barrier, v) of the excitonic transistor. Color scale indicated normalized PL intensity. **b** (i) Control of exciton concentration using an excitonic transistor with h-BN spacer. (ii) Stark shift as a function of applied vertical electric field (${\mathop {\textit{E}}\limits ^{\rightarrow }}_{z}$) in a heterotrilayer device (Device A) and a heterobilayer device (Device B). Real-space PL images corresponding to anti-confinement (iii), free diffusion (iv), and confinement (v) behaviors of IXs. (vi) Peak emission energy inside and outside the gate region as a function of applied electric field, with different incident power levels represented by red and blue curves. (vii) Interlayer exciton density as a function of applied electric field, with different incident power levels shown by red and blue curves. **c** (i) IX diode and transistor featuring etched FLG top gates incorporating a triangular slide structure. (ii) COSMOL calculation depicting the vertical electric field component beneath the etched graphene region. (iii) CCD images capturing laser intensity (left), gates off state (middle), and gates on state (right). **a** Reproduced with permission [40]. Copyright [2018] Springer Nature. **b** Reproduced with permission [41]. Copyright [2019] Springer Nature. **c** Reproduced with permission [42]. Copyright [2022] Americal Chemical Society

While IXs do not carry a net electric charge, recent studies demonstrated the spatial control of IXs using out-of-plane electric field [40–43]. In the 2D TMD heterostructures, IXs have spatially separated holes and electrons within different layers, resulting in a large out-of-plane dipole moment *p* [44]. Consequently, an electric field *E*$_{z}$(*x*, *y*) perpendicular to the crystal plane enables the modulation of IXs energy $\Delta$ $\varepsilon$ through quantum confined Stark effect with the relation, $\Delta$ $\varepsilon$ $=$ $-$ ${\mathop {\textit{p}}\limits ^{\rightarrow }}$ $\cdot$ ${\mathop {\textit{E}}\limits ^{\rightarrow }}$ [45]. This modification of IX energy can be used to transport the IXs, as IX current flows toward the low energy.

Employing this quantum confined Stark effect, the recent study by Unuchek et al. demonstrated an excitonic transistor at room temperature, as illustrated in Fig. 3a-(i) [40]. Without applying an electric field (Fig. 3a-(ii)), when the excitation light was focused on the 2D TMD heterostructures, IXs were generated and diffused along the channel of the transistor by both the concentration of IXs and temperature [26, 46, 47]. By contrast, applying electric field induced the potential barrier higher than *k*$_{\text {B}}$*T* in the diffusion path, blocking the channel of the transistor, as shown in Fig. 3a-(iii). Therefore, this procedure effectively facilitated the operation of excitonic transistor, as shown in Fig. 3a-(iv-v). These findings revealed the capability of 2D TMD heterostructures in electrically controlling the spatial distribution of excitonic quasiparticles, paving the way toward IX-based excitonic devices [41–43].

The local application of the electric field enabled the artificial electrostatic traps, achieving the high exciton concentration [41]. Figure 3b-(i) illustrated the 2D TMD heterostructures, with a monolayer h-BN spacer. The spacer effectively reduced moiré potential limiting diffusion/drift of IXs [48–51] while increasing the separation between electrons and holes enhancing the magnitude of the out-of-plane dipole moment ${\mathop {\textit{p}}\limits ^{\rightarrow }}$ of IXs. Figure 3b-(ii) showed the quantum confined Stark shift as a function of the applied electric field in two devices with (device A) and without the spacer (device B). The dipole size *d* was 0.9 nm and 0.6 nm in each device. The difference between two devices was similar to the size of a monolayer h-BN. Here, the device A exhibited larger energy shift compared to the device B as the slope of the energy shift was proportional to the magnitude of the out-of-plane dipole moment ${\mathop {\textit{p}}\limits ^{\rightarrow }}$ of IXs. Figure 3b-(iii-v) showed the electrical control of the IX distribution by the local generation of the potential well, clearly representing the confinement and anti-confinement of IX distribution compared to their natural diffusion. The specific density of IXs can be estimated from the nonlinear energy shift of IXs as a function of the applied electric field (Fig. 3b-(vi)), attributed to the quantum confined Stark effect and the exciton–exciton interaction:

1 $$\begin{aligned} \begin{array}{cl} \Delta \varepsilon = -{\mathop {\textit{p}}\limits ^{\rightarrow }}\cdot {\mathop {\textit{E}}\limits ^{\rightarrow }} + \frac{n_{\text {IX}}de^2}{\varepsilon _{\text {HZ}}\varepsilon _0} \end{array} \end{aligned}$$,

where $n_{\text {IX}}$ is maximum exciton density, $\varepsilon _{\text {HZ}}$ $=$ 6.26 is the effective relative permittivity of the heterotrilayer, and $\varepsilon$ $_0$ is the vacuum permittivity. The nonlinear component in Fig. 3b-(vii) was solely originated from the change in IX density. Therefore, the IX density with applying electric field can be derived with the Eq. 1, as shown in Fig. 3b-(vii). This result facilitates the high concentration of IXs, potentially leading to strong repulsive dipole-dipole interactions, enabling long-range IXs transport [52]. The high concentration of IXs harnesses the potential to produce a degenerate Bose gas with the capability to control the concentration of polarized excitons and realize the high-temperature Bose-Einstein condensation of excitons [35, 53, 54].

While previous studies demonstrated the devices enabling radial IX transport, the recent study by Shanks et al. presented a specialized device facilitating unidirectional IX transport, more suitable for excitonic-circuit applications, as shown in Fig. 3c-(i) [42]. The patterned graphene top gate generated the gradient of the electric field, leading to the gradual decrease in the IX bandgap energy through the patterned area, as shown in Fig. 3c-(ii). Figure 3c-(iii) showed the spatially resolved PL images when the manipulated electric field was applied, demonstrating the electrically switchable unidirectional flow of the IX current. In this study, they manipulated IXs transport utilizing a highly directional IX diode with a customizable channel. This novel excitonic device can lead to the low-power, high-speed IX circuits operable at room temperature.

In this section, we have reviewed recent advancements in the electrical control of exciton transport. Unlike the limited transport observed in neutral excitons in TMD monolayers, negatively charged excitons and IXs can be electrically transported. This capacity for electrical transport enables applications in industrial circuits [55]. By utilizing various devices using charged excitons and IXs, we can achieve not only long-range propagation lengths but also extended lifetimes [37, 38, 56, 57]. Future work will involve designing highly integrated IX devices to enhance the controllability of IX transport [58]. This development will pave the way for various conventional excitonic devices [4, 23, 59].

## Strain control for exciton transport

**Fig. 4:**
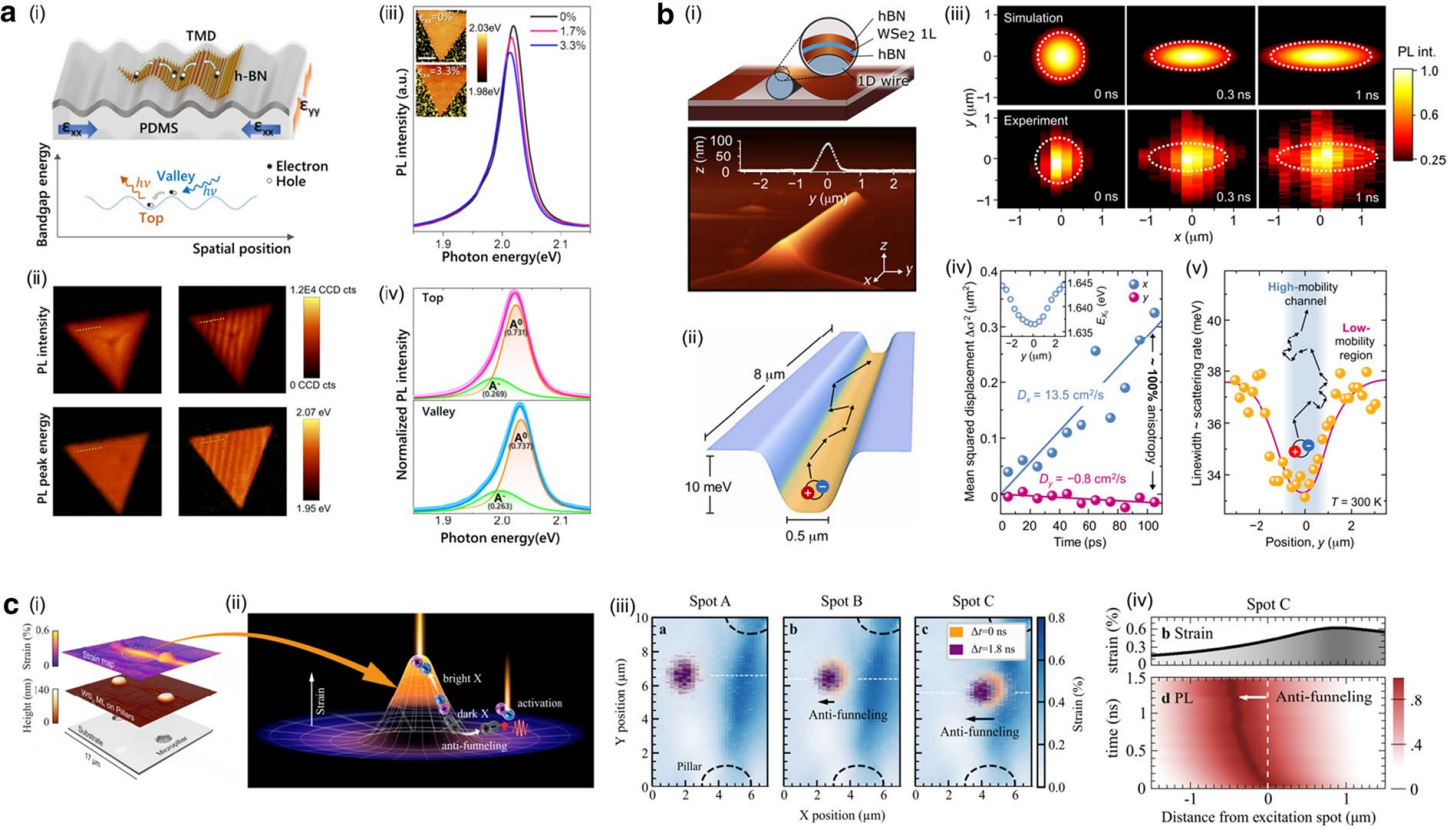
**a** (i) Illustration depicting a wrinkled WS$_2$ monolayer and its corresponding energy diagram. (ii) PL intensity and energy image of a single WS$_2$ monolayer crystal. (iii) PL spectra obtained under different tensile strains of 0 $\%$, 1.7 $\%$, and 3.3 $\%$. (iv) Fitted PL spectra acquired at the top (top) and valley (bottom) regions of the wrinkled WS$_2$ monolayer. **b** (i) AFM topography image showing a WSe$_2$ monolayer transferred onto a nanowire. (ii) Illustration demonstrating the behavior of photo-generated excitons diffusing along a quasi-1D channel. (iii) Spatially and temporally resolved distribution of photogenerated excitons, obtained through simulation (top) and experiment (bottom). (iv) Displacement of excitons in the x-axis and y-axis as a function of time. (v) Linewidth profile demonstrating spatially varying intervalley exciton–phonon scattering rate. **(c)** (i) Topography image (middle panel), along with an applied strain map (top panel), showing a WS$_2$ monolayer transferred onto a micropillar. (ii) Illustration highlighting the behaviors of bright excitons and dark excitons in the presence of strain gradient. (iii) Spatially and temporally resolved distribution of dark excitons at different positions (spot A, B, and C), corresponding to distinct locations within the strain gradient region. (iv) Detailed time-dependent behavior analysis focusing on dark excitons at spot C. **a** Reproduced with permission [60]. Copyright [2022] Americal Chemical Society. **b** Reproduced with permission [61]. Copyright [2021] AAAS. **c** Reproduced with permission [62]. Copyright [2021] Springer Nature

TMDs have remarkable sustainability to the applied strain [63] with exciton stability at room temperature [1, 64], which provides unique platforms for continuously modifying the electronic and optical characteristics. The generation of strain-gradient leads to the local modification of the exciton density, as the exciton current flows toward a low bandgap energy. Therefore, various strain-gradient geometries have been suggested for the specific applications, such as harvesting the photon energy as a form of excitons and controlling the exciton current for the exciton integrated circuits [19, 60, 61, 65–67].

Recently, Lee et al. suggested the tunable device enabling the controlled wrinkle formation of WS$_2$ monolayer, as shown in Fig. 4a-(i) [60]. The tensile strain can be applied at the top position of the wrinkle, which decreases the bandgap energy. This led the photo-generated excitons to be effectively confined at the top of the wrinkle, as shown in Fig. 4a-(ii). To confirm that the local enhancement of the PL intensity was due to the exciton funneling effect, the collective PL spectra were obtained under increasing tensile strain. As the tensile strain is known to decrease both PL energy and intensity, the redshifted PL spectra with the decreased emission intensity were observed with the application of the tensile strain, as shown in Fig. 4a-(iii). In addition, the negligible spectral changes of PL spectra obtained at the top and valley of the wrinkle demonstrated the insignificant role of the charged exciton formation while reconfirming the major role of the exciton funneling effect, as shown in Fig. 4a-(iv). These works clearly present the strain-induced exciton funneling effect in WS$_2$ monolayers with systematic characterizations including PL energy and intensity under various circumstance of the tensile strain. The large dimension of their strain-induced optical tunability over the whole crystal provides capability to develop tunable optoelectronic devices or exciton circuits in TMD monolayers [23].

While previous study presented the one-dimensional (1D) confinement of the photo-generated excitons in WS$_2$ monolayer, Dirnberger et al. recently demonstrated the directional exciton flow with enhanced mobility through locally suppressed exciton–phonon scattering in 1D strain gradient geometry [61]. Figure 4b-(i) showed the atomic force microscopy (AFM) image of the WSe$_2$ monolayer on the nanowire, inducing the decrease in the bandgap energy along the nanowire. This generated the quasi-1D channel for the exciton diffusion, as shown in Fig. 4b-(ii). Figure 4b-(iii) demonstrated the temporally-resolved exciton distributions when the excitons were injected at the center of the potential well. As shown in Fig. 4b-(iv), the exiton flow exhibited significant anisotropy along the nanowire axis, while presenting enhanced mobility compared to the unstrained cases. At room temperature, Fig. 4b-(v) clearly showed the reduction of intervalley exciton–phonon scattering at the quasi-1D channel, leading to the high-mobility channel for the confined excitons. This works provided an understanding of exciton movement at 1D strained geometry, leading to the development of excitonic architectures and networks.

While the bright excitons exhibit the funneling toward the minima of tensile stain, the dark excitons drift away from the minima of tensile strain, showing the anti-funneling effect. This is attributed to the opposite stain-induced bandgap energy variation of dark excitons, as the energy of momentum-dark excitons increases with applying tensile strain [62, 68]. Recently, Rosati et al. demonstrated the anti-funneling effect of dark excitons in WS$_2$ monolayer, strained on the micropillar, as shown in Fig. 4c-(i-ii) [62]. To confirm the anti-funneling effect of dark excitons, the spatial-temporal PL images were obtained at different positions. As the excitation spot moved toward higher stain gradient, the temporally resolved anti-funneling of dark excitons were observed, as shown in Fig. 4c-(iii). Specifically, with increasing strain gradient, dark excitons drifted from the original position to the lower strained area with higher funneling velocity. Figure 4c-(iv) the spatially-temporally resolved PL intensity at the highest strain gradient, demonstrating clear anti-funneling effect. This study investigated the movement of dark excitons under the strain gradient, which showed the completely different nature to the bright excitons. The anti-funneling effect possesses the capability to develop efficient energy harvesting system. The key aspect of developing efficient photovoltaics relies on the efficiency of photocurrent generation from exciton dissociation, which is significantly affected by contact properties [62]. The inverse funneling system allows a flexibility of electrode design, which enhances the quality of the contact.

**Fig. 5:**
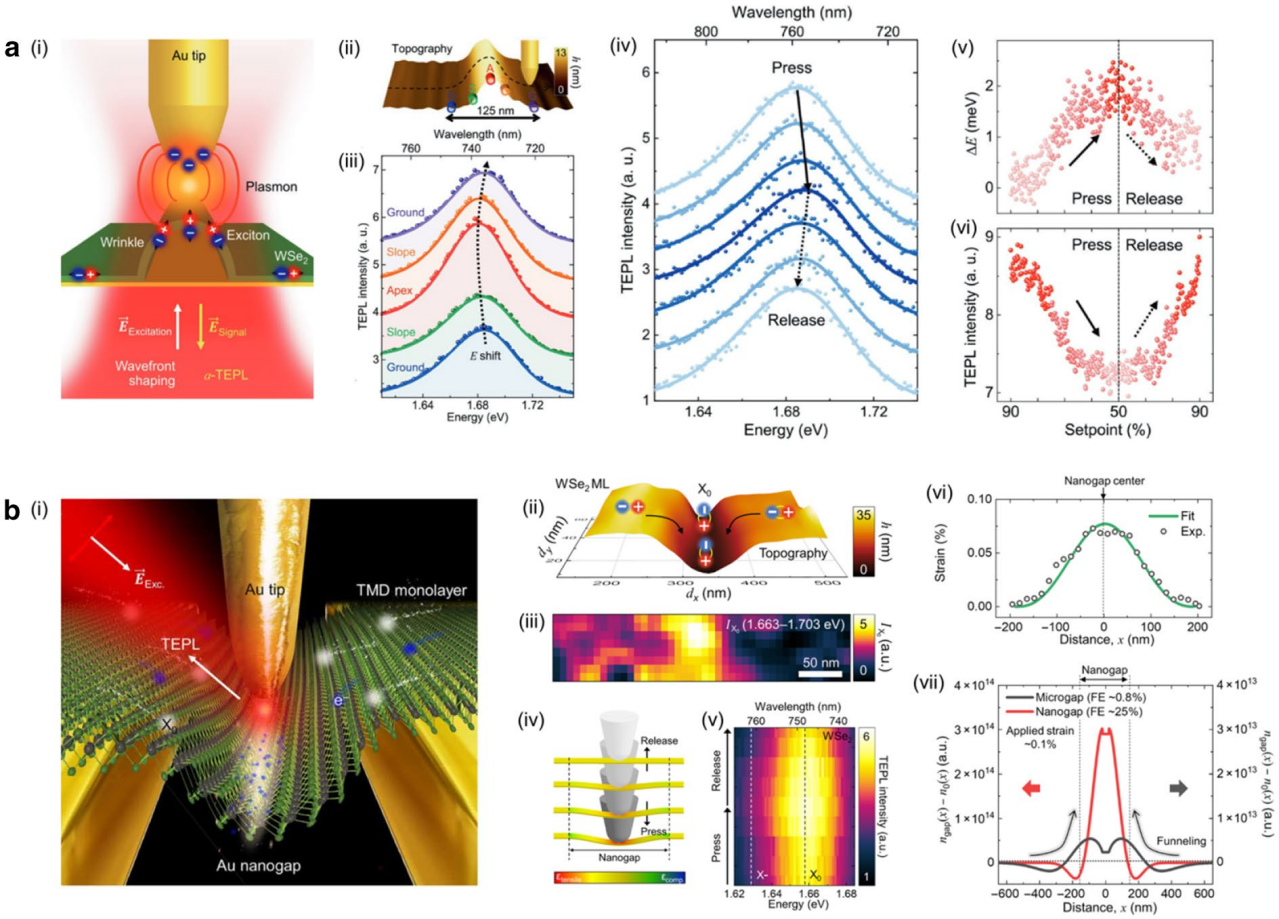
**a** (i) Illustration of TEPL spectroscopy probing nanoscale wrinkle in WSe$_2$ monolayer. Topography profile revealing a nanoscale wrinkle (ii), and its corresponding TEPL spectra obtained at the wrinkle site (iii). (iv) TEPL spectra acquired as the tip pressed against and released from the apex of the nanoscale wrinkle. Reversible control of TEPL energy (v) and intensity (vi) by changing tip position, i.e., pressure on the wrinkle apex. **b** (i) Illustration depicting a WSe$_2$ monolayer transferred onto a nanogap structure, investigated using TEPL spectroscopy. Topography image showing the surface morphology of the WSe$_2$ monolayer on the nanogap structure (ii), accompanied by corresponding TEPL intensity mapping (iii). (iv) Illustration demonstrating tip-induced modification of strain gradient within the WSe$_2$ monolayer region near the nanogap structure. (v) Reversible control of TEPL intensity facilitated by dynamic control of tip position. (vi) Estimated strain gradient for suspended WSe$_2$ monolayer on nanogap. (vii) Theoretical estimation of exciton distribution under microscale strain gradient (black curve) and nanoscale strain gradient (red curve). **a** Reproduced with permission [69]. Copyright [2021] John Wiley and Sons. **b** Reproduced with permission [70]. Copyright [2022] AAAS

When the strain gradient scales down to the nanoscale, different characteristics of exciton transport with a new controllability can be driven in TMD monolayers. However, the nanoscale strain gradient induced by sub-diffraction-limited geometry significantly limits the optical investigation with conventional optical microscopy technique. Recently, the development of tip-enhanced photoluminescence (TEPL) spectroscopy enables the investigation of nano-optical phenomena with its sub-diffraction-limited spatial resolution [69–74].

The recent study by Koo et al. presented the applications of nanoscale wrinkles in WSe$_2$ monolayers as bright quantum-nanophotonic devices, as illustrated in Fig. 5a-(i) [69]. While the wrinkles in as-grown and transferred TMD monolayers were inevitable heterogeneities, their sub-diffraction-limited size of $\sim$100 nm significantly restricted the comprehensive understanding of their optical characteristics. Employing TEPL spectroscopy allowed them to investigate optical properties of nanoscale heterogeneities by obtaining spatially resolved TEPL spectra. Figure 5a-(ii) showed the topography image of the nanoscale wrinkle, demonstrating its nanoscale width of $\sim$125 nm. The TEPL spectra were then obtained across the wrinkle, i.e., at the ground, slope, and apex of the wrinkle, as shown in Fig. 5a-(iii). As the measurement spot moved from the ground to the apex of the wrinkle, the TEPL spectra showed increased intensity with decreased emission energy, clearly demonstrating the exciton funneling effect. In addition, the plasmonic tip of TEPL spectroscopy facilitated the direct mechanical modulation of the wrinkle structure, indicating the nano-optical controllability of the exciton funneling magnitude. Indeed, Fig. 5a-(iv) showed the TEPL spectra obtained by reversibly applying the pressure on the top of wrinkle structure. As the tip pressed the wrinkle, the decreased TEPL intensity with increased emission energy was observed, attributed to the reduced exciton funneling effect by the compressive strain. When the tip was retracted, the TEPL intensity and emission energy turned back to the original values, confirming the reversible engineering, as shown in Fig. 5-(v-vi). These findings pave the way toward investigating nano-optical phenomena related to the nanoscale heterogeneities in TMD monolayers [75, 76]. The presented reversible controllability on the emission intensity and energy at the nanoscale region provides opportunities to develop nano-light-emitting diodes (nano-LEDs) and nano-optical switches.

More recently, Lee et al. reported the in-depth investigation of exciton funneling effect at the nanoscale strain gradient [70]. They combined WSe$_2$ monolayer with the nanogap geometry to deterministically induced the nanoscale strain gradient, which was indeed an inverse geometry of nanoscale wrinkle. Then, employing TEPL spectroscopy facilitated to spatially resolve nanoscale optical response at the nanoscale strain gradient at the nanogap, as illustrated at Fig. 5b-(i). Figure 5b-(ii) showed the topography image of the WSe$_2$ monolayer transferred on the nanogap geometry, generating nanoscale strain gradient with the width of $\sim$200-300 nm. Due to the exciton funneling effect, the enhanced emission of WSe$_2$ excitons was observed at the center of the nanogap, as shown in Fig. 5b-(iii). Figure 5b-(iv-v) demonstrated the reversible control of exciton funneling effect through the dynamic positioning of plasmonic tip in TEPL spectroscopy. Interestingly, the exciton confinement by the exciton funneling effect revealed much stronger at nanoscale strain gradient compared to the microscale gradient. Indeed, analyzing drift-diffusion equation with experimentally obtained strain gradient profile demonstrated the higher funneling efficiency at the nanoscale strain gradient, as shown in Fig. 5b-(vi-vii). This was attributed to the drift-dominant nature of the nanoscale strain gradient, as the drift current significantly affected by the gradient of the applied strain. As a result, they achieved $\sim$25 $\%$ of funneling efficiency with just $\sim$0.1 $\%$ of applied strain at room temperature, which much exceeded the $\sim$1 $\%$ of funneling efficiency with $\sim$2 $\%$ of applied strain in the previous study [19]. These findings provide comprehensive understanding of the exciton funneling effect at the nanoscale strain gradient, exhibiting pronounced differences to the microscale strain gradient. Achieving the high funneling efficiency at room temperature is the building block of developing highly efficient excitonic photovoltaics [77] and exciton integrated circuits [23]. The more optimized design of strain gradient geometry further enhances the funneling efficiency, decreasing the thermal loss of exciton flow at room temperature.

In this section, we have reviewed recent progresses to control exciton flow in TMD monolayers with strain gradient geometry. By exploiting the various strain gradient geometry and different optical characteristics of excitonic quasiparticles, the correspondingly modified behaviors of exciton flow can be optimized to the specific applications, such as energy harvesting in the excitonic photovoltaics [77] and exciton transport in the exciton–integrated circuits [23]. In future, designing the optimized strain gradient geometry and employing the complex bandgap structure of IX in TMD heterostructures [4] are expected to further enhance the exciton funneling efficiency.

## Coupling with surface plasmon polaritons for exciton transport

**Fig. 6:**
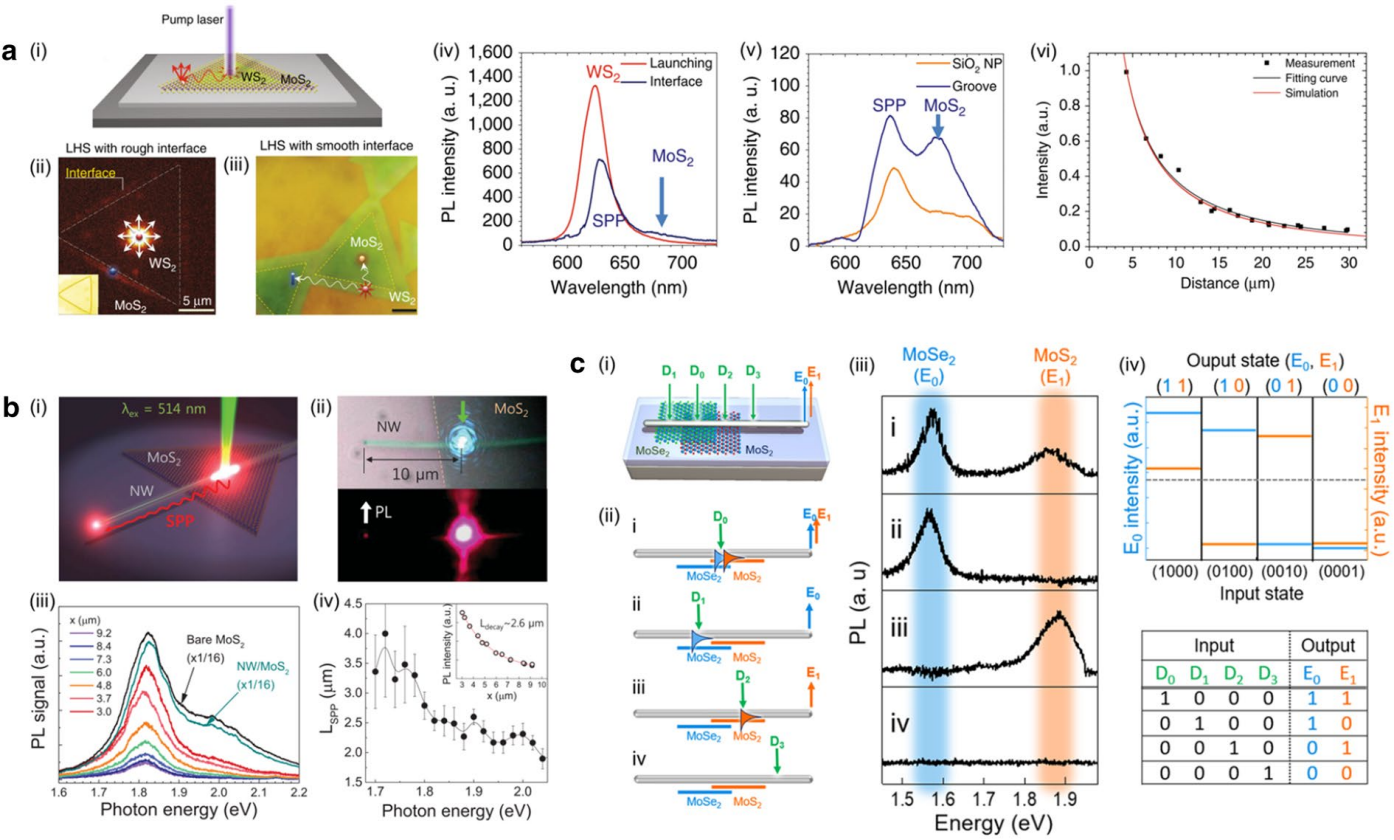
**a** (i) Schematic representation of the exciton transfer process occurring in a monolayer WS$_2$/MoS$_2$ heterostructure transferred onto an Ag plate. (ii) PL image of the lateral heterostructure. Excitons in the WS$_2$ monolayer (depicted as red dots) were coupled to SPP through a non-radiative decay process. Subsequently, SPPs induced excitons in the MoS$_2$ monolayer at the interface (depicted as blue dots). (iii) Optical microscopy images of the WS$_2$/MoS$_2$ TMD lateral heterostructure. Excitons were generated in the WS$_2$ monolayer at the laser spot (indicated by a red star). PL spectra were collected from regions on the SiO$_2$ nanoparticle (marked as an orange dot) and groove on the silver plate (indicated by a blue slit). (iv) PL spectra collected at the launching point and interface as shown in (ii). (v) PL spectra collected in the SiO$_2$ nanoparticle and groove in the MoS$_2$ region as shown in (iii). (vi) Variation of PL intensity observed during the exciton–SPP–exciton conversion process as a function of propagation. **b** (i) Schematic illustration demonstrating the propagation of exciton-coupled SPPs. MoS$_2$ excitons were generated by a 514 nm wavelength laser at the overlapping region where the nanowire intersected the MoS$_2$ monolayer. (ii) Top :  Optical micrograph depicting the Ag nanowire and MoS$_2$ flake with the incident laser spot. Bottom :  PL image showing the end of the Ag nanowire and the MoS$_2$ monolayer. (iii) PL spectra collected at the end of the nanowire, with variations in excitation positions. (iv) SPP propagation lengths determined from PL spectra in relation to photon energy. Inset :  Peak of PL intensity as a function of transport length *x*. **c** (i) Schematic of an Ag nanowire positioned on a partially stacked MoSe$_2$/MoS$_2$ bilayer with laser illuminations at four terminals. Output signals are collected at the right end of the Ag nanowire. (ii) Four combinations of excitation positions on the MoSe$_2$/MoS$_2$ monolayer. (iii) Collected PL spectra corresponding to the different combinations of light illuminations at the input terminals. (iv) Implementation of an optical 4-to-2 binary encoder through plotting the measured intensity as a function of the four different input states. **a** Reproduced with permission [78]. Copyright [2017] Springer Nature. **b** Reproduced with permission [79]. Copyright [2015] John Wiley and Sons. **c** Reproduced with permission [80]. Copyright [2019] Springer Nature

The exciton transport as a coupled form with SPP exhibits the potential for the development of plasmonic optical interconnects and circuits [81–85]. The plasmon-exciton coupling enables the long-range transport of exciton emission [78, 81, 86–89] while maintaining intriguing optical characteristics of 2D TMDs, such as valley degree of freedom and indirect bandgap lasing [64, 82, 84, 90–93]. In addition, coupling to plasmon leads to the concentration of the electromagnetic fields down to the sub-diffraction-limit, enhancing the degree of integration in circuit applications [78, 81–83, 94–97].

The long-range exciton transport mediated by the SPP has been facilitated by exploiting cascaded exciton energy transfer within a lateral TMD heterostructure, as shown in Fig. 6a-(i) [78]. Figure 6a-(ii) showed the PL image with rough interface conditions facilitating the formation of SPP through a non-radiative process on the Ag plate. The excitons in WS$_2$ monolayer generated by an incident laser generated the SPP by the rough interface, subsequently generated the excitons of MoS$_2$ monolayer at the interface. As shown in Fig. 6a-(iv), while excitons were produced in the WS$_2$ monolayer, excitons of MoS$_2$ monolayer were detected at the interface of the WS$_2$ and MoS$_2$ monolayer. By contrast, smoothing the interface broke the energy transfer barrier at the interface. In this regime, deterministic positioning of the cascaded exciton energy transfer spot can be realized with the groove and the SiO$_2$ nanoparticle due to the translational symmetry breaking or local hot SPP spots, as shown in Fig. 6a-(iii). Figure 6a-(v) clearly demonstrated the MoS$_2$ emission at the groove and SiO$_2$ nanoparticle, mediated by the plasmonic transport of excitons of the WS$_2$ monolayer. The detailed propagation length of these cascade exciton–SPP–exciton energy transfer was shown in Fig. 6a-(vi). They demonstrated the observation of the exciton emission nearly $\sim$40 $\mu$m away from the excitation spot, which sufficiently overcame the intrinsic exciton diffusion length of TMDs. These findings highlighted the simpler fabrication process to enable longer exciton transport compared to the traditional methods, while plasmonic substrate significantly enhanced the emission intensity for harvesting exciton emission with the high efficiency.

While previous study relied on the directionless propagation of substrate-induced SPP, the directional propagation of exciton emission has been demonstrated through the SPP of the Ag nanowire, as shown in Fig. 6b-(i) [79]. When the incident laser focused on the overlapping region of the Ag nanowire and MoS$_2$ monolayer, the generated excitons of MoS$_2$ monolayer were directly coupled to the SPP of Ag nanowire. The coupled exciton emission propagated through the Ag nanowire and emitted exciton emission at the end of the Ag nanowire. The top panel of Fig. 6b-(ii) showed the optical micrograph of the Ag nanowire and the MoS$_2$ flake with the incident laser spot, while the bottom panel exhibited the PL image at the end of the Ag nanowire and the excitation spot. The PL emission decreased with increasing excitation distance from the end of the Ag nanowire, yet SPP-coupled exciton can be clearly observed until the distance of $\sim$10 $\mu$m, as shown in Fig. 6b-(iii). Figure 6b-(iv) showed the relationship between the SPP propagation length and the photon energy, demonstrating the larger reabsorption and Ohmic loss of the high-energy emission. In the case of MoS$_2$ emission, the PL intensities derived by fitting to the exponential decay function decreased exponentially as a function of distance demonstrated the parameter of $\sim$2.6 $\mu$m. This study suggests the potential for integrating plasmonic waveguides with TMD materials to achieve directional excitonic transport by exploiting the unique characteristics of TMDs, such as highly bounded excitons at room temperature and distinctive valley properties.

Exciton-based optical logic operation was performed using the Ag nanowire on the stacked MoSe$_2$/MoS$_2$ monolayers [80]. In this study, they demonstrated several logic operations, including AND, XOR, and 4-to-2 binary encoder operations. Specifically, Fig. 6c-(i) showed the schematic of the 4-to-2 binary encoder operation, which was one of the logic operations. By changing the incident laser spot on the Ag nanowire, excitons in MoSe$_2$ and MoS$_2$ monolayer were selectively generated and propagated, as shown in Fig. 6c-(ii). Four terminals were determined depending on the crystal locations: D0 on the bilayer, D1 on the MoSe$_2$ monolayer, D2 on the MoS$_2$ monolayer, and D3 without TMDs. The transport signal was then collected at the end of the Ag nanowire. Figure 6c-(iii) showed the output states, which are determined by PL spectra of MoSe$_2$/MoS$_2$ excitons corresponding to input terminals. The PL intensities corresponding to the exciton energy of TMDs were shown in Fig. 6c-(iv). The dashed line served as a criterion to determine output states as 0 or 1, which are described in the bottom table. These results demonstrated the potential for the optical nanoprocessors and the integrable excitonic circuits.

In this section, we have reviewed the exciton transport with the SPP of the plasmonic structures. SPP enables not only the overcoming of the intrinsic exciton transport length through the cascaded exciton–SPP–exciton transfer but also directional exciton propagation by coupling to the plasmonic waveguide. To effectively harness the advantages of plasmonic transport, increasing the coupling and conversion efficiency while minimizing the undesirable losses during the propagation plays an important role. Plasmonic structures possess a large degree of freedom in geometry-dependent optical and electrical properties, providing opportunities to develop new design of SPP-mediated exciton transport platform.

## Photonic cavity for exciton transport

**Fig. 7:**
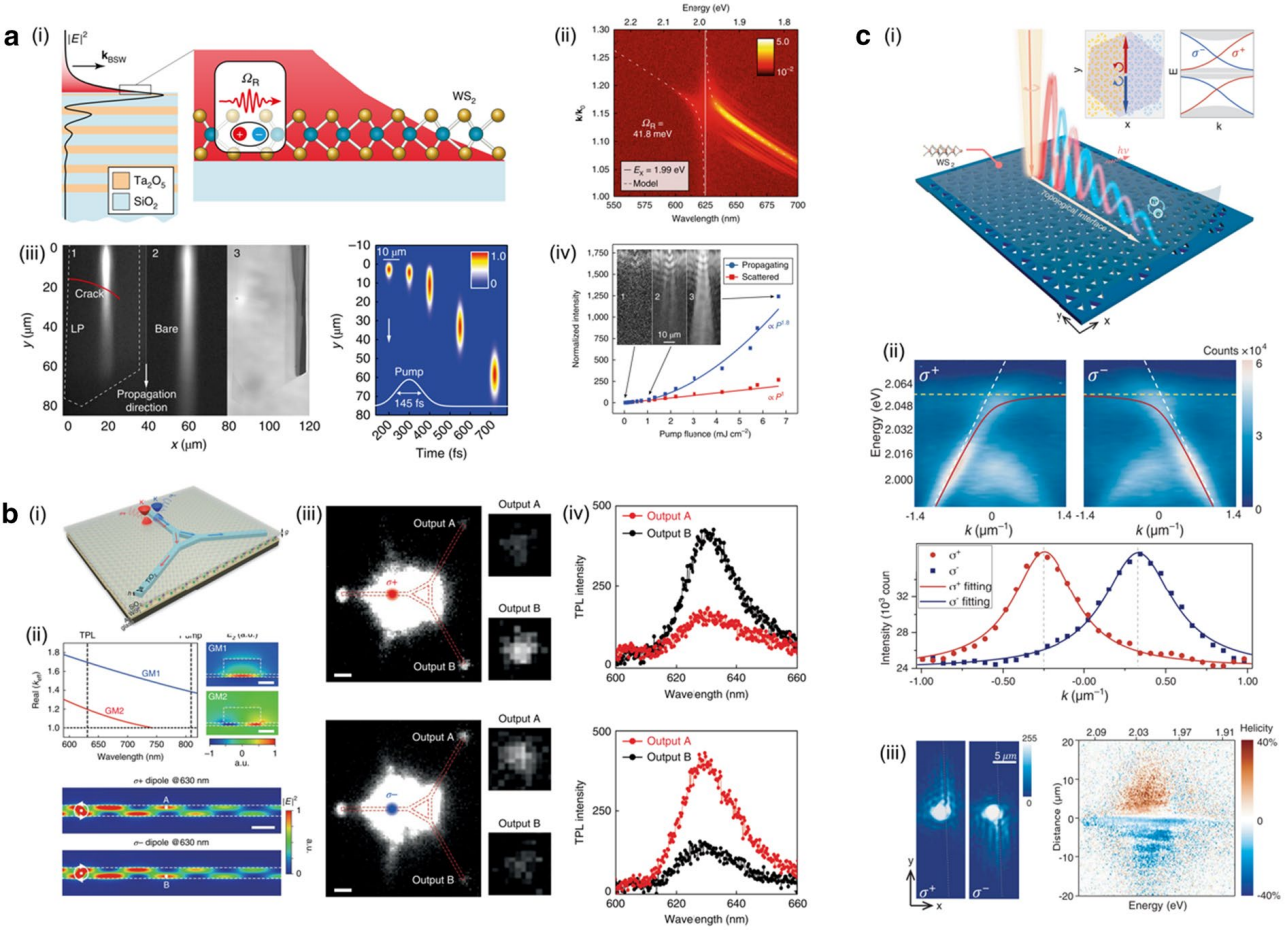
**a** (i) Schematic diagram illustrating the coupling between BSW at the surface of the stack and in-plane excitons in the monolayer WS$_2$. (ii) Experimental dispersion observed in the PL spectrum. (iii) Time-resolved image capturing the propagation of BSW polaritons. (iv) PL intensity plot demonstrating nonlinear power-dependence. **b** (i) Illustration of the nanocircuit enabling the direction routing of K and K$^{\prime }$ valley-dependent emission from the WS_2_ monolayer. (ii) Numerical simulations depicting gap waveguide modes, showcasing the principle of valley preservation. (iii) Two-photon photoluminescence (TPL) images of the valley router when excited by a focused pump laser with $\sigma +$ or $\sigma$- polarization. (iv) TPL spectra measured from ports A and B for $\sigma +$ or $\sigma$- polarized excitations. **c** (i) The topological polaritonic system comprising a monolayer WS_2_ strongly coupled to a nontrivial photonic crystal. (ii) Angle-resolved PL spectra of the helical interface polaritons with $\sigma +$ and $\sigma$- polarized light emissions at the domain wall. (iii) Real-space image capturing the propagation of helical polaritons along the topological domain wall with $\sigma +$ and $\sigma$- excitation. (iv) Spectral and spatial characterization of the helicity of interface polariton propagation, with the color scale representing the degree of helicity. **a** Reproduced with permission [98]. Copyright [2018] Springer Nature. **b** Reproduced with permission [99]. Copyright [2022] Springer Nature. **c** Reproduced with permission [100]. Copyright [2020] AAAS

Photonic systems are primarily characterized by their optical responses based on key degrees of freedom, which include frequency, wavevector, polarization, and phase. For several decades, ongoing research efforts have been dedicated to facilitating optical systems with predetermined optical attributes through meticulous selection of materials, precise manipulation of structures, and deliberate control of dimensions. By integrating a custom-designed photonic cavity structure considering the exciton resonances of 2D materials, it becomes feasible to couple excitons with specific photonic modes. This capability has various applications in exciton transport, such as extending the propagation distance [78, 86, 87, 98, 101, 102] and enabling directional propagation of exciton emission without applying external stimulus [79, 99, 103]. In addition to the conventional methods, recent attempts have been increasing to utilize new degree of freedom of photonic systems, such as chirality [99, 104–106] and topology [100, 107, 108].

When the strong coupling is achieved between excitons and cavity photons, a novel bosonic quasiparticle is generated, known as exciton–polaritons [109, 110]. This entity assumes a distinctive form, inheriting characteristics, such as lighter effective mass compared to excitons, while retaining the cavity photon’s lifetime, and featuring a potent binding energy superior to that of excitons [111–113]. These attributes enable the propagation of waves with distinct characteristics in both photonic and excitonic transport. The generation of exciton–polariton has been demonstrated using a range of different schemes and mechanisms, including high-Q cavities with distributed Bragg reflectors (DBRs) [86, 114–119], photonic crystals [102, 103, 105, 120, 121], and plasmonic nanocavities [122–124], and hybrid cavities [98, 102, 125, 126]. Recently, Barachati et al. suggested the two-dimensional polaritonic exciton transport method by combining DBR structure and TMD monolayer [98]. A-excitons of WS$_2$ monolayer were strongly coupled to Bloch surface waves (BSW) emerged at the surface of Ta$_2$O$_5$/SiO$_2$ DBR structure, as illustrated in Fig. 7a-(i). Formation of BSW-exciton polaritons was verified by the anti-crossing around the exciton energy, as shown in Fig. 7a-(ii). For quantitative analysis of propagation characteristics of BSW polaritons, time-resolved real space image for lower polariton branch energy was taken, as presented in Fig. 7a-(iii). Consequently, propagation was observed over $\sim$60 $\mu$m which was more than 10 times longer than the diffusion length of neutral excitons in TMD monolayer at room temperature [18, 47]. Moreover, Fig. 7a-(iv) exhibited the nonlinearly increased intensity of propagating polaritons to the excitation power, which arose from the peak shift of polariton branches depending on the coupling strength of BSW and WS$_2$ excitons. These findings highlighted the potential of the long-range exciton–polaritonic transport system through the incorporation of the designed structure. Since the system possessed excellent tunability, this approach can be applied to the various two-dimensional materials. However, the directionless movement of exciton polariton along the flake limited the capability of effectively controlling the direction of exciton transport, necessary to facilitate practical applications.

Valley pseudospin of electrons in hexagonal 2D materials arose from the inequivalent electronic properties at the band edge of +K and −K. By controlling the chirality of incident photons, selective exciton formation from the +K and −K valley became possible [127]. In a recent investigation conducted by Chen et al. introduced a method for waveguiding and routing valley-selective exciton emission [99]. Figure 7b-(i) illustrated a TiO$_2$ nanocircuit on an SiO$_2$/WS$_2$/Au structure designed to directionally route two-photon PL (TPL) from valley excitons in the WS$_2$ monolayer using the chirality of the incident photons. The principle behind maintaining valley-dependent excitonic properties of WS$_2$ was demonstrated in Fig. 7b-(ii). Two strongly confined propagating gap modes were formed by the coupling between TiO$_2$ waveguide modes and the SPP of the gold surface. At the wavelength of TPL, a beating pattern was created due to the wavevector mismatch of the two gap modes, as simulated in Fig. 7b-(iii). Considering the in-plane circular dipoles $\sigma$- and $\sigma +$ , which correspond to the circularly polarized TPL emission originating from the K$^{\prime }$ and K valleys of the monolayer WS$_2$, the interference pattern displayed a contrasting profile for the $\sigma$- and $\sigma +$ dipoles. This difference in the spatial distribution enabled valley-dependent routing when the positions of the routing paths were precisely controlled. Figure 7b-(iv) provided an optical microscope image, which showed the incident laser spot and the success of the spin-dependent routing via output ports A and B, as confirmed by the accompanying PL spectra. These results suggested unique method of preserving valley properties even when coupled with propagating waveguide modes. In an exciton transport perspective, these findings offered the significant scalability over existing structures and held the crucial potential for applications, such as all-optical excitonic transistors and logic circuits.

Since the discovery of topological insulators, the topology has become an active area of research in condensed matter systems and has extended into the realm of photonics [128–131]. The topology in photonics provides unique features, including back-scattering-free transport properties, compactness, and robustness. Recently, a photonic crystal structure was designed to exhibit the quantum spin Hall phase which enabled chirality-dependent exciton–polariton transport through a topological interface, as shown in Fig. 7c-(i) [100]. The structure consisted of a WS$_2$ monolayer on two topologically distinct hexagonal lattice sections, with each unit cell comprised of six triangular holes. The topology of the lattice was controlled by arranging the positions of the holes, as the intercell and intracell coupling strengths changed accordingly. As A-excitons of the WS$_2$ monolayer were strongly coupled to the topological interface in the photonic lattice, bulk polariton bands and pseudospin-dependent Dirac-type topological interface polariton bands were formed. The helical nature of the topological interface states originated from the opposite signs of angular momenta for pseudospin-up and pseudospin-down states, corresponding to the circular polarization of the incident beams $\sigma$- and $\sigma +$. Figure 7c-(ii) displayed the E-k dispersion curve and the profile of the topological interface polariton modes, with opposite signs of group velocity depending on the excitation polarization. As a result, selective unidirectional propagation of helical interface polaritons in a specific direction depending on the excitation polarization was enabled and directly visualized through real-space imaging, as shown in Fig. 7c-(iii). Moreover, spatial and spectral characteristics were investigated by extracting the degree of circular polarization of the interface polariton emission as a function of photon energy and propagation distance, as depicted in Fig. 7c-(iv). In this study, a new degree of freedom, topology, has been successfully introduced into exciton–photonic crystal hybrid structure to generate topological exciton polaritons in two-dimensional boundary and chirality dependent path-selective unidirectional propagation was achieved. These investigations opened up a new avenue for active topology control in polaritonic systems. The design techniques used in the literature have great potential to be applied to other topological structures, such as quantum valley Hall states [132] and higher-order states [133]. As a result, a wide range of high-quality excitonic transport mechanisms can be tailored for specific purposes by coupling various topological states with excitons.

In this section, we have reviewed recent studies of photonic structures designed to couple with excitons in two-dimensional materials. By harnessing the rich excitonic properties of TMDs, various structures including DBR, dielectric-metal hybrid gap waveguide, and topological photonic crystal have been utilized to achieve outcomes, such as low-loss, long-range transport, unidirectional propagation, and efficient routing. These results serve as essential prerequisites for practical applications of exciton-based devices, such as optoelectronic interconnects and photonic circuit elements. Given the substantial potential and ample room for improvement in two-dimensional exciton transport through the integration of photonic structures, we anticipate the emergence of more innovative and progressive research in this field.

## Conclusion

In this comprehensive review, we have explored the recent progress in the control of exciton transport in various strategies, with a particular focus on its applications in 2D semiconductors. To overcome the lack of controllability for neutral excitons, the short lifetime of charged excitons, and the low exciton funneling efficiency, we explored how incorporating newly developed control parameters of excitonic quasiparticles and structural advancement have attributed to their progress in the field of exciton-based devices. Throughout the review, we delved into four control parameters for excitonic quasiparticle flow in two-dimensional semiconductors.

First, we discussed about manipulation of excitonic quasiparticles driven by electric fields. Adjusting the electric field on two-dimensional semiconductors allowed to control the behavior of charged excitons within the circuit. Our exploration extended to interlayer charged excitons, distinguished by their prolonged lifetimes, which enabled the propagation of excitonic quasiparticles in a longer distance. Furthermore, neutral interlayer exciton flow can be driven by the electric field, by the modulation of interlayer exciton energy through quantum confined Stark effect.

Second, we explored the strain control of neutral excitons via strain gradient in two-dimensional semiconductors on the artificial structures. Strain gradient induced the local modification of the exciton density, as the exciton current flowed toward a low bandgap energy, allowing neutral excitons propelled along the shape of artificial structure. Meanwhile, we introduced the anti-funneling effect under the strain gradient by exploiting the opposite strain-induced energy variation of momentum-dark excitons. Furthermore, nanoscale strain gradients were revealed to possess discernable characteristics such as drift-dominant exciton flow for significantly enhancing the exciton funneling efficiency at room temperature.

Third, our investigation centered on the examination of long-range exciton transport facilitated by SPP within planar two-dimensional semiconductors. The coupling of excitons to plasmons served to concentrate electromagnetic fields at sub-diffraction-limited scale, thereby amplifying the potential for integration within electronic circuits. Additionally, exciton–SPP complexes can be directionally propagated through coupling with plasmonic waveguides.

Lastly, we examined exciton transport coupled with specific photonic modes within photonic cavity structures. By coupling various photonic cavity structures, considering the exciton resonances of two-dimensional semiconductors, it led to excitons coupling with specific photonic modes. This phenomenon facilitated the realization of low-loss and long-range exciton transport. Additionally, it enabled unidirectional propagation via controlling chirality of incident photons, consequently leading to the selective formation of excitons from the +K and −K valleys.

In this review, while we have primarily discussed the platforms for controlling exciton transport, it is important to acknowledge that various strategies have been concurrently developed to further improve both the speed and the degree of control over exciton transport. For example, exploiting moiré excitons in twisted heterostructures introduced interesting phenomena originated from modified local atomic configurations and diffusivities [50], such as three distinct phases of interlayer excitons depending on their densities [134] and non-linear transport phenomena by interacting with higher-order charge complexes [135]. In addition, the recent study by Águila et al. demonstrated the ultrafast and long-range transport of exciton fluid in MoS$_2$ monolayer, extending its potential to circuit operations [136]. While this approach was currently limited to low-temperature operation, their strategies for harnessing new phases of excitons offered an alternative pathway for developing a practical exciton transport modality.

In addition to the exciton transport in monolayer and bilayer two-dimensional semiconductors, exciton transport can be applied to the transport of single photon emitters combined with waveguide structure [137, 138]. Furthermore, exciton transport can be extended to near-infrared and infrared region by exploiting new materials combined to appropriate cavity structures, broadening its applications to optical communications and networks.

## Data Availability

Not applicable
